# Pan-cancer multi-omics analysis of pharmacogenomic alterations

**DOI:** 10.3389/fbinf.2026.1858518

**Published:** 2026-07-22

**Authors:** Aisha AlMulla, Simerpreet Kaur, Prachi Balyan, Sana Al-Saafin, Manoj Kumar Balyan, Dinesh Velayutham, Puthen Veettil Jithesh

**Affiliations:** 1 College of Health and Life Sciences, Hamad Bin Khalifa University (HBKU), Qatar Foundation, Doha, Qatar; 2 Division of Genetics, Department of Pediatrics, All India Institute of Medical Sciences, New Delhi, India; 3 CSIR-Centre for Cellular and Molecular Biology, Hyderabad, India; 4 Qatar Precision Health Institute, Qatar Foundation, Doha, Qatar; 5 Pharmacology and Therapeutics, Institute of Systems, Molecular and Integrative Biology, University of Liverpool, Liverpool, United Kingdom

**Keywords:** copy number alteration, multi-omics integration, pan-cancer analysis, pharmacogenomics, precision oncology

## Abstract

**Background:**

Pharmacogenomic (PGx) variation is an important determinant of inter-individual variability in drug response. While germline pharmacogenomics has been extensively studied, somatic alterations affecting these pharmacogenes in tumors remain less systematically characterised.

**Methods:**

We performed a pan-cancer multi-omics analysis integrating somatic mutation, copy number alteration, and transcriptomic data across 765 patients with primary tumors spanning seven The Cancer Genome Atlas (TCGA) cancer types. Exploratory survival analyses were restricted to 733 patients with complete clinical and overall survival information. Using a curated panel of 40 pharmacogenes, we characterized gene-level alterations, summarised recurrent events across tumor types, and aggregated these signals into pharmacological modules representing key pharmacological processes.

**Results:**

Pharmacogene alterations were generally infrequent at the individual gene level but showed recurrent, non-uniform patterns across cancers. A uniform-gene null model showed that descriptive combined scores were more concentrated than expected under a simplified uniform-gene background, with a global Gini index of 0.306 compared with a null mean of 0.248, and the top 10 gene-cancer pairs accounting for 10.1% of the total score compared with a null mean of 7.8%. Broad non-synonymous mutation, copy number amplification, and copy number deletion frequencies averaged 3.8%, 4.7%, and 0.3%, respectively, while the mean frequency of any integrated alteration was 8.6%. A descriptive prioritization score, defined as broad non-synonymous mutation frequency plus copy number amplification frequency, identified recurrent high-ranking alterations in transporter- and drug-handling genes, including *ABCB1*, *ABCC1*, and *ABCC2*. Sensitivity analyses supported cautious interpretation, including partial ranking robustness across stricter truncating/LoF-like mutation definitions, small-cohort exclusions, and cBioPortal GISTIC amplification sensitivity analyses. Systematic CNA-expression testing across 280 gene-cancer pairs identified 64 significant positive associations after FDR correction, with representative examples including *GGH*, *ERCC1*, and *SLC29A1*. Module-level pharmacogenomic alteration was not significantly associated with survival in exploratory treatment-agnostic survival models.

**Conclusion:**

Our findings provide an exploratory TCGA-based map of somatic alterations of pharmacogenes across cancers and identify recurrent alteration patterns that warrant validation in treatment-annotated and external datasets.

## Introduction

Pharmacogenomics provides a framework for understanding variability in drug response by linking genetic variation to pharmacokinetic and pharmacodynamic processes (Relling and Evans, 2015; Jithesh et al., 2022). In oncology, this framework is particularly relevant because of the complexity of treatment regimens, the narrow therapeutic index of many agents, and the frequent emergence of drug resistance. While germline pharmacogenomic variation has been incorporated into clinical decision-making for selected drugs, somatic alterations within tumors represent an additional and less well-characterized source of variability that may influence treatment outcomes (Tsimberidou et al., 2020).

Tumor genomes are characterized by extensive molecular heterogeneity, including somatic mutations, copy number alterations, and transcriptional dysregulation. Large-scale initiatives such as The Cancer Genome Atlas (TCGA) have enabled systematic characterization of these alterations across multiple cancer types, providing a major resource for pan-cancer analyses (Cancer Genome Atlas Research Network et al., 2013). Integrated pan-cancer studies have further shown that molecular patterns often reflect shared processes that extend beyond tissue of origin (Hoadley et al., 2018; Sanchez-Vega et al., 2018). However, most such studies have focused primarily on oncogenic drivers rather than pharmacogenes, leaving the somatic pharmacogenomic landscape across cancers less clearly defined. Recent reviews have also emphasized the role of bioinformatics resources, variant annotation frameworks, multi-omics integration, and computational prediction models in advancing pharmacogenomics toward personalized medicine (Zaid et al., 2026).

Pharmacogenes encode proteins involved in drug transport, metabolism, detoxification, and DNA damage repair, and their dysregulation may alter tumor-associated drug handling. For example, ATP-binding cassette (ABC) transporters play a central role in multidrug resistance by mediating drug efflux (Fletcher et al., 2010; Muriithi et al., 2020), while DNA repair genes such as *ERCC1* influence sensitivity to platinum-based therapies (Olaussen et al., 2006; Lord and Ashworth, 2012). The integration of multi-omics data offers an opportunity to more comprehensively characterize pharmacogenomic alterations across tumor types. By combining mutation, copy number, and expression data, it is possible to capture complementary mechanisms of gene dysregulation and identify patterns that would not be apparent from single-layer analyses. Furthermore, the integration of clinical data enables assessment of the prognostic and potential predictive relevance of these alterations.

In this study, we performed a pan-cancer multi-omics analysis of somatic pharmacogene alterations across seven TCGA cancer types. We aimed to characterize the landscape of pharmacogene alterations, identify recurrent and non-uniform patterns at both the gene and module levels, evaluate copy number-associated expression concordance, and perform exploratory treatment-agnostic survival analyses. Given the absence of harmonized treatment exposure and response data, the study was framed as an exploratory molecular research rather than a direct assessment of pharmacogenomic actionability.

## Methods

### Study cohort and data acquisition

This study was designed as a retrospective TCGA pan-cancer multi-omics analysis of somatic alterations in pharmacogenes using publicly available data (Table 1). Molecular alteration analyses were performed in 765 patients with harmonized mutation, copy number, and RNA-sequencing data across seven TCGA cancer types: breast invasive carcinoma (TCGA-BRCA), colon adenocarcinoma (TCGA-COAD), liver hepatocellular carcinoma (TCGA-LIHC), lung adenocarcinoma (TCGA-LUAD), ovarian serous cystadenocarcinoma (TCGA-OV), rectum adenocarcinoma (TCGA-READ), and stomach adenocarcinoma (TCGA-STAD). Survival analyses were restricted to 733 patients with complete overall survival information and clinical covariates, comprising 127 TCGA-BRCA, 120 TCGA-COAD, 87 TCGA-LIHC, 187 TCGA-LUAD, 52 TCGA-OV, 31 TCGA-READ, and 129 TCGA-STAD patients. Multi-omics datasets, including somatic mutation, gene expression, gene-level copy number scores, and clinical annotations, were accessed through the Genomic Data Commons using TCGAbiolinks 2.38.0 (Colaprico et al., 2016) in R 4.5.2/Bioconductor 3.22.

**TABLE 1:** Molecular analysis cohort and survival-analysis subset across TCGA cancer types.

Project	Tumor type	Molecular multi-omics cohort, n	Survival/Clinical subset, n	Excluded from survival subset, n	Median age (years)	Male (%)	Female (%)
TCGA-BRCA	Breast	133	127	6	59.9 (50.0–70.0)	2.4	97.6
TCGA-COAD	Colon	130	120	10	69.1 (60.9–77.6)	50.8	49.2
TCGA-LIHC	Liver	88	87	1	62.9 (54.8–69.0)	74.7	25.3
TCGA-LUAD	Lung	190	187	3	65.3 (58.0–71.3)	50.3	49.7
TCGA-OV	Ovarian	52	52	0	59.9 (55.4–71.8)	0.0	100.0
TCGA-READ	Rectal	32	31	1	65.5 (57.8–71.8)	64.5	35.5
TCGA-STAD	Stomach	140	129	11	69.6 (61.6–75.3)	59.7	40.3
Total		765	733	32	65.5 (56.7–72.9)	43.7	56.3

Molecular alteration frequencies were calculated using the harmonized molecular multi-omics cohort. Survival analyses were restricted to the clinical subset with complete overall survival and covariate data. TCGA, the cancer genome atlas.

### Pharmacogene panel and functional modules

A curated pharmacogene panel comprising 40 genes was assembled based on established roles in anticancer drug transport, metabolism, detoxification, nucleotide/folate biology, and DNA damage repair. Gene selection was informed by pharmacogenomics databases and literature, including PharmGKB and Clinical Pharmacogenetics Implementation Consortium resources (Hewett, 2002; Relling and Klein, 2011). Genes were assigned to five pharmacological modules: fluoropyrimidine, antimetabolite, irinotecan, platinum, and transporter-related. Because several pharmacogenes participate in more than one pharmacological process, genes were permitted to contribute to multiple modules where appropriate. These modules were used as transparent analytical groupings rather than drug-specific clinical prediction models. The gene-level panel rationale is provided in Table 2, with module definitions and gene-to-module membership provided in Supplementary Tables S1, S2.

**TABLE 2:** Curated pharmacogene panel, functional category, module assignment, and inclusion rationale. Representative drug relevance and module membership are shown for each gene. Genes were allowed to contribute to more than one module where appropriate. Module definitions and gene-to-module membership are provided in Supplementary Tables S1, S2.

Gene	Functional category	Representative drug relevance	Analysis module assignment	Inclusion rationale
*ABCB1*	ABC transporter/drug efflux	Taxanes; irinotecan; multiple xenobiotics	Transporter-related; Irinotecan	Included as a major efflux transporter implicated in intracellular drug disposition and multidrug resistance.
*ABCC1*	ABC transporter/drug efflux	Platinum agents; multiple xenobiotics	Transporter-related; Platinum	Included as a drug transporter with relevance to efflux of anticancer drugs and detoxification-related transport.
*ABCC2*	ABC transporter/drug efflux	Irinotecan; platinum agents; multiple xenobiotics	Transporter-related; Irinotecan; Platinum	Included because of its role in hepatobiliary/drug transport and anticancer drug handling.
*ABCC3*	ABC transporter/drug efflux	Multiple anticancer drugs and xenobiotics	Transporter-related	Included as part of the transporter network relevant to drug disposition.
*ABCG2*	ABC transporter/drug efflux	Irinotecan; TKIs; multiple substrates	Transporter-related; Irinotecan	Included as a broad substrate transporter relevant to drug efflux and resistance.
*AOX1*	Xenobiotic metabolism/detoxification	Multiple xenobiotics	Antimetabolite	Included as a xenobiotic metabolism gene with relevance to drug biotransformation.
*CDA*	Nucleoside metabolism	Gemcitabine; cytarabine; antimetabolites	Antimetabolite; Fluoropyrimidine	Included because nucleoside metabolism can affect antimetabolite exposure and activity.
*CES1*	Carboxylesterase/prodrug metabolism	Irinotecan	Irinotecan	Included for its role in carboxylesterase-mediated drug activation/metabolism.
*CES2*	Carboxylesterase/prodrug metabolism	Irinotecan	Irinotecan	Included because CES2 is involved in irinotecan/SN-38-related metabolism.
*CYP1A1*	Cytochrome P450 metabolism	Carcinogens; selected anticancer/xenobiotic substrates	Antimetabolite	Included as a CYP-mediated xenobiotic metabolism gene.
*CYP1A2*	Cytochrome P450 metabolism	Selected TKIs and xenobiotic substrates	Antimetabolite	Included as a CYP-mediated xenobiotic metabolism gene.
*CYP2C19*	Cytochrome P450 metabolism	Cyclophosphamide and other substrates	Antimetabolite	Included as a clinically relevant CYP pharmacogene.
*CYP2C8*	Cytochrome P450 metabolism	Paclitaxel and other substrates	Antimetabolite	Included as a CYP pharmacogene relevant to anticancer drug metabolism.
*CYP2C9*	Cytochrome P450 metabolism	Cyclophosphamide and other substrates	Antimetabolite	Included as a CYP pharmacogene relevant to drug metabolism.
*CYP2D6*	Cytochrome P450 metabolism	Tamoxifen and other substrates	Antimetabolite	Included as a well-established pharmacogene with anticancer drug relevance.
*CYP3A4*	Cytochrome P450 metabolism	Taxanes; TKIs; multiple drugs	Antimetabolite	Included as a major CYP enzyme involved in metabolism of multiple anticancer agents.
*CYP3A5*	Cytochrome P450 metabolism	Taxanes and multiple drugs	Antimetabolite	Included as a CYP3A family pharmacogene relevant to drug metabolism.
*DCK*	Nucleoside activation	Cytarabine; gemcitabine	Antimetabolite	Included because nucleoside activation is central to antimetabolite efficacy.
*DPYD*	Fluoropyrimidine catabolism	5-FU; capecitabine	Fluoropyrimidine; Antimetabolite	Included as a core fluoropyrimidine pharmacogene.
*ERCC1*	DNA repair/nucleotide excision repair	Platinum agents	Platinum	Included because DNA repair capacity is relevant to platinum response.
*ERCC2*	DNA repair/nucleotide excision repair	Platinum agents	Platinum	Included as a DNA repair gene relevant to platinum-associated DNA damage response.
*FPGS*	Folate pathway/polyglutamation	Methotrexate and antifolates	Antimetabolite	Included because folate pathway regulation can affect antifolate response.
*GGH*	Folate pathway/polyglutamation	Methotrexate and antifolates	Antimetabolite	Included because folate pathway regulation can affect antifolate exposure and activity.
*GSTP1*	Detoxification/glutathione conjugation	Platinum agents and xenobiotics	Platinum	Included because detoxification pathways can influence platinum and xenobiotic handling.
*MTHFR*	Folate pathway	Methotrexate; 5-FU-related folate metabolism	Fluoropyrimidine; Antimetabolite	Included as a folate-pathway pharmacogene relevant to antimetabolite biology.
*NT5C2*	Nucleotide metabolism	Thiopurines; antimetabolites	Antimetabolite	Included because nucleotide metabolism can influence antimetabolite activity.
*NUDT15*	Thiopurine metabolism	Thiopurines	Antimetabolite	Included as a clinically relevant thiopurine pharmacogene.
*RRM1*	Ribonucleotide reductase	Gemcitabine	Antimetabolite	Included because ribonucleotide metabolism is relevant to gemcitabine and antimetabolite response.
*SLC22A1*	Solute carrier transporter	Platinum agents; TKIs; organic cations	Transporter-related; Platinum	Included as an uptake transporter relevant to intracellular drug exposure.
*SLC22A2*	Solute carrier transporter	Cisplatin and organic cations	Transporter-related; Platinum	Included as an uptake transporter relevant to platinum handling.
*SLC22A3*	Solute carrier transporter	Organic cations; multiple drugs	Transporter-related; Platinum	Included as part of the uptake transporter network.
*SLC28A1*	Nucleoside transporter	Antimetabolites	Transporter-related; Antimetabolite	Included because nucleoside transport influences antimetabolite uptake.
*SLC29A1*	Equilibrative nucleoside transporter	Gemcitabine; nucleoside analogues	Transporter-related; Antimetabolite	Included because nucleoside transport is central to gemcitabine uptake.
*SLCO1B1*	Organic anion transporter	Methotrexate and multiple drugs	Transporter-related; Antimetabolite	Included as a hepatic/drug uptake transporter.
*SLCO1B3*	Organic anion transporter	Taxanes and multiple drugs	Transporter-related	Included as a transporter relevant to drug disposition.
*TPMT*	Thiopurine metabolism	Thiopurines	Antimetabolite	Included as a clinically established thiopurine pharmacogene.
*TYMP*	Pyrimidine metabolism	Capecitabine; fluoropyrimidines	Fluoropyrimidine; Antimetabolite	Included because pyrimidine metabolism is relevant to fluoropyrimidine biology.
*TYMS*	Thymidylate synthase	5-FU; fluoropyrimidines	Fluoropyrimidine; Antimetabolite	Included as a core fluoropyrimidine target/pathway gene.
*UGT1A1*	Glucuronidation/detoxification	Irinotecan	Irinotecan	Included as a core irinotecan pharmacogene involved in SN-38 glucuronidation.
*XRCC1*	DNA repair/base excision repair	Platinum agents	Platinum	Included as a DNA repair gene relevant to DNA damage response.

### Multi-omics preprocessing and integration

Gene expression data were obtained from TCGA using the STAR-Counts workflow. Gene-level counts were extracted from SummarizedExperiment objects, restricted to the predefined pharmacogene panel, and transformed as:

$$E_{ig}=\log_{2}\left({count}_{ig}+1\right)$$

where *E*_*ig*_ denotes the transformed expression value for gene *g* in sample *i.*

Within each cancer type, gene expression values were standardized to z-scores for each gene:

$$z_{ig}^{expr}=\frac{E_{ig}-\mu_{g,c}}{\sigma_{g,c}}$$

where $E_{ig}$ is expression of gene g in sample i , and $\mu_{g,c}$ , $\sigma_{g,c}$ are the mean and standard deviation within cancer type c . Expression dysregulation was defined as overexpression: $z_{ig}^{expr}>2$ and underexpression: $z_{ig}^{expr}<-2$

Gene-level copy number data were likewise restricted to the pharmacogene panel. After harmonization with matched RNA and mutation data, copy number values were standardized gene-wise within each cancer type as:

$$z_{ig}^{cna}=\frac{C_{ig}-\mu_{g,c}^{cna}}{\sigma_{g,c}^{cna}}$$

Where $C_{ig}$ is the copy number value for gene *g* in sample *i*, and $\mu_{g,c}^{cna}$ and $\sigma_{g,c}^{cna}$ are the corresponding mean and standard deviation within cancer type *c*. Strict copy number amplification and deletion states were defined as $z_{ig}^{cna}>2$ and $z_{ig}^{cna}<-2$ , respectively. Softer gain and loss states, defined as $z_{ig}^{cna}>1$ and $z_{ig}^{cna}<-1$ , were retained for descriptive visualisation only and were not used in the primary integrated alteration definition.

Somatic mutation data were obtained from TCGA masked mutation annotation format files generated using the MuTect2 pipeline. Mutations were filtered to include only the curated pharmacogene panel. To avoid overinterpreting missense variants as functionally damaging, variants were classified into separate mutation categories. Broad non-synonymous mutations included missense, nonsense, splice-site, frameshift, translation-start-site, nonstop, and in-frame insertion/deletion variants. Truncating/LoF-like mutations included nonsense, frameshift, splice-site, and translation-start-site variants. Missense mutations and in-frame indels were also retained as separate categories for sensitivity analyses. Binary mutation matrices were constructed per gene and patient.

Sample identifiers were harmonized using TCGA barcode conventions, with 16-character sample barcodes used for molecular data and 12-character patient barcodes used for clinical integration. For each cancer type, only genes and samples shared across RNA expression, copy number, and mutation layers were retained. An integrated binary alteration flag was then defined for each gene and sample as:

$$A_{ig}=I\left(M_{ig}^{nsm}=1\vee C{NA}_{ig}^{amp}=1\vee {CNA}_{ig}^{del}=1\right)$$

Where $A_{ig}$ represents the integrated alteration state for gene *g* in sample *i*. $M_{ig}^{nsm}$ indicates the presence of a broad non-synonymous mutation, $C{NA}_{ig}^{amp}$ ​ indicates strict amplification, and ${CNA}_{ig}^{del}$ indicates strict deletion. Additional concordance variables were derived to capture alignment between copy number state and expression direction, including amplification with overexpression and deletion with under-expression.

### Gene-level summarization and prioritization

For each cancer type and pharmacogene, alteration frequencies were summarized across the integrated cohort, including frequencies of any mutation, broad non-synonymous mutation, truncating mutation, strict amplification, strict deletion, overexpression, underexpression, any integrated alteration, and aligned multi-omic events. To prioritize recurrent gene-cancer pairs descriptively, a combined prioritization score was calculated as the sum of broad non-synonymous mutation frequency and strict copy number amplification frequency. This score was used only as a descriptive ranking index and was not interpreted as a functional or clinically actionable score.

$$S_{g,c}=f_{g,c}^{nsm}+f_{g,c}^{amp}$$

where $S_{g,c}$ is the descriptive score for gene *g* in cancer type $f_{g,c}^{nsm}$ ​ is the broad non-synonymous mutation frequency, and $f_{g,c}^{amp}$ is the strict amplification frequency.

To evaluate the sensitivity of gene-cancer rankings to mutation definition, prioritization analyses were repeated using stricter truncating/LoF-like mutations and amplification-only rankings. Ranking overlap was calculated between the broad non-synonymous, truncating/LoF-like, and amplification-only top 30 gene-cancer pairs. To assess the influence of small cohorts, prioritization analyses were also repeated after excluding cohorts with fewer than 50 and fewer than 100 molecularly profiled patients.

Because the primary copy-number analysis used cancer-type-specific z-score thresholds, we performed a sensitivity analysis using thresholded cBioPortal TCGA PanCancer Atlas GISTIC copy-number calls. For this analysis, GISTIC values were interpreted as −2 deep deletion, −1 shallow deletion, 0 neutral, +1 gain, and +2 amplification. Strict GISTIC amplification was defined as +2, and broad gain/amplification was defined as +1 or +2. Gene-cancer rankings based on strict GISTIC amplification were compared with the primary z-score-based CNA rankings.

### Uniform-gene null model for alteration-score concentration

To evaluate whether pharmacogene alteration scores were more concentrated than expected under a simplified uniform-gene model, we performed 5,000 null simulations. Within each cancer type, the total number of broad non-synonymous mutation events and copy number amplification events was preserved, but events were randomly redistributed across the 40-gene pharmacogene panel with equal gene probability. For each simulation, descriptive combined scores were recalculated and compared with the observed score distribution. Concentration metrics included the Gini index, maximum combined score, and the proportion of total score contributed by the top 10 and top 30 gene-cancer pairs. Empirical p-values were calculated as the proportion of null simulations with values equal to or greater than the observed value.

### Module scoring

Module-level scores were computed to summarize pharmacogenomic alteration burden within each pathway. Module burden was defined as the number of genes within a module showing an integrated alteration:

$${\text{ModuleBurden}}_{im}=\sum_{g\in m}I\left(A_{ig}=1\right)$$

where $A_{ig}$ is the integrated alteration flag for gene *g* in sample *i*, and *m* denotes the set of genes in each module.

In addition, module proportion was defined as the mean integrated alteration status across all genes within a module:

$${ModuleProp}_{im}=\frac{1}{\left|m\right|}\sum_{g\epsilon m}A_{ig}$$

Thus, module burden reflects the absolute number of altered genes in a pathway, whereas module proportion reflects the fraction of genes altered within that pathway. Module burden was used for descriptive visualization, while module proportion was used for cohort-level summaries and the primary survival models.

To avoid selective interpretation of representative copy number-expression examples, CNA-expression concordance was tested systematically across all pharmacogene-cancer pairs. For each gene-cancer pair, Spearman correlation was used to evaluate the association between copy number z-score and expression z-score. Linear models were also fitted using expression z-score as the outcome and copy number z-score as the predictor. Where sufficient samples were available, expression distributions were compared between copy number states. P-values were adjusted across all tested gene-cancer pairs using the Benjamini–Hochberg false discovery rate method. Representative examples for Figure 5 were selected from significant CNA-expression associations identified in this systematic analysis.

### Survival analyses

Clinical data were standardized across projects using patient-level barcodes. Overall survival (OS) time was defined using days to death when available and otherwise days to last follow-up or days to last known alive. Event status was derived from vital status. Patients without usable follow-up time were excluded from survival analyses. Associations between pharmacogenomic module alteration and overall survival were assessed using Cox proportional hazards models. For each module, the primary model used module proportion as a continuous predictor standardized per standard deviation. Age at diagnosis and sex were included as covariates when available, and cancer type was incorporated as a stratification factor. The model can be written as:

$$h_{i}\left(t\right)=h_{0k}\left(t\right)\exp\left(\beta_{1}X_{i}^{*}+\beta_{2}{Age}_{i}+\beta_{3}{Sex}_{i}\right)$$

where $h_{i}\left(t\right)$ is the hazard for patient *i*, $h_{0k}\left(t\right)$ is the baseline hazard for stratum *k* defined by cancer type, and $X_{i}^{*}$ ​ is the standardized module proportion value for the corresponding module. Kaplan-Meier curves were generated for visualization using the corresponding median-split grouping. Cox proportional hazards models were fitted separately for each module burden score, with age and sex included as covariates and cancer type used as a stratification factor.

All analyses were conducted using R (4.5.2) and Bioconductor (3.22) with packages including TCGAbiolinks (2.38.0), maftools (2.26.0), tidyverse (2.0.0), ComplexHeatmap (2.26.1), and survival (3.8–6) and survminer (Colaprico et al., 2016; Mayakonda et al., 2018; Wickham et al., 2019; Gu, 2022; Kassambara et al., 2026; Therneau et al., 2026). Scripts were organized into modular workflows covering data acquisition, preprocessing, integration, and analysis. Heatmaps of alteration frequencies were generated using the ComplexHeatmap package. Expression distributions were visualized using boxplots, and survival curves were generated using survminer (0.5.2).

## Results

### Pan-cancer pharmacogenomic alteration landscape

Across the 40-gene pharmacogene panel and seven TCGA cancer types, molecular alteration analyses were performed in 765 patients with harmonized mutation, copy number, and expression data. Gene-level pharmacogene alterations were generally infrequent when considered individually, but their distribution was non-uniform across cancer types and genes. Mean frequencies across all gene-cancer combinations were 3.8% for broad non-synonymous mutations, 4.7% for copy number amplification, and 0.3% for copy number deletion, whereas the mean frequency of any integrated alteration was 8.6%. Deletion events were particularly sparse, with a median deletion frequency of zero across most gene-cancer pairs, indicating that deletion contributed minimally to the overall landscape. By contrast, amplification showed a broader distribution and represented the dominant recurrent genomic alteration within the panel. Maximum broad non-synonymous mutation frequency reached 25%, maximum amplification frequency reached 13.1%, and the maximum frequency of any integrated alteration reached 31.3%, indicating that a relatively small subset of pharmacogenes accounted for a disproportionate share of the observed signal.

**FIGURE 1:**
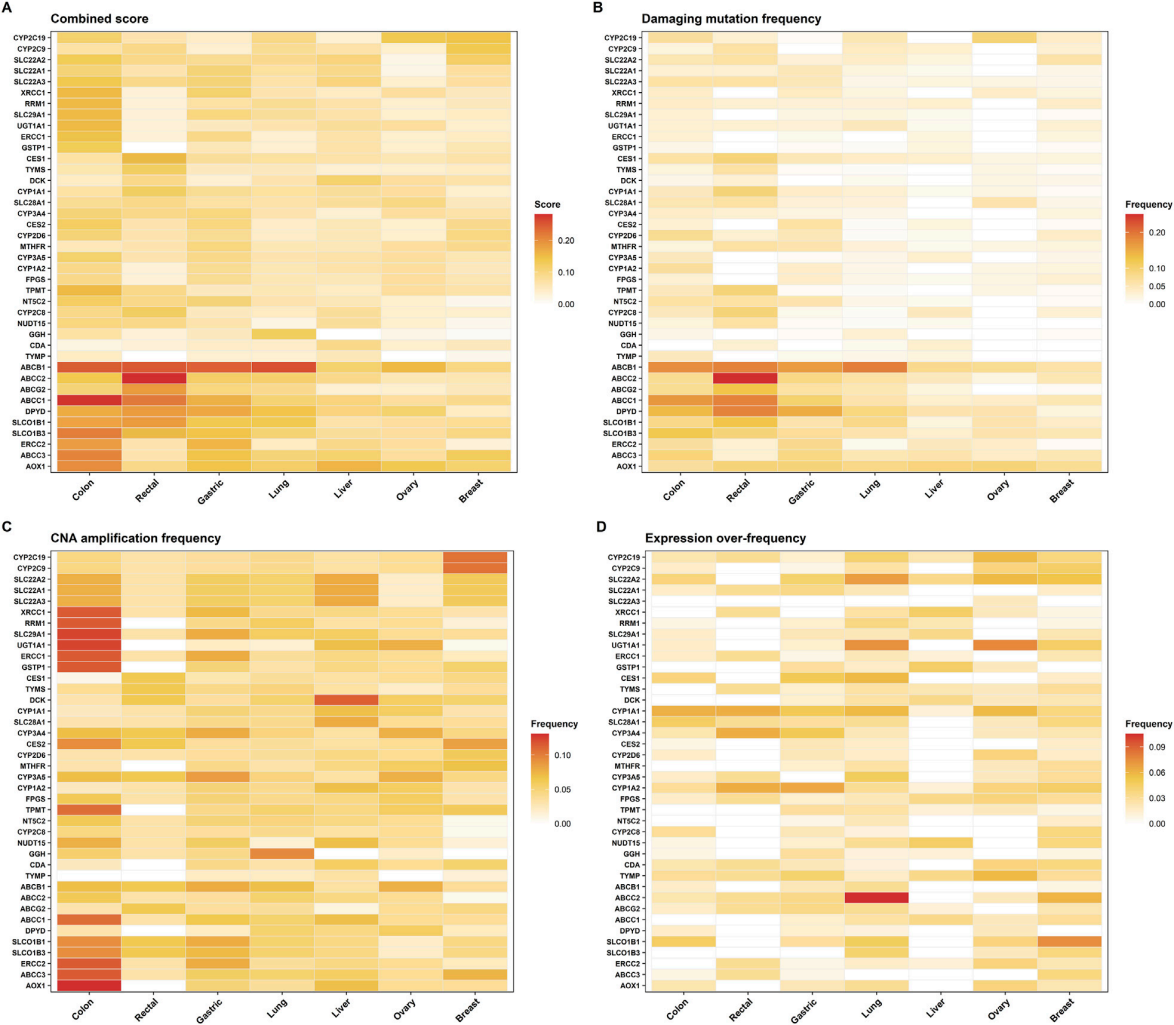
Pan-cancer landscape of pharmacogenomic alterations across molecular layers. Across all panels, rows represent pharmacogenes and columns represent cancer types. Genes are displayed in a consistent order across panels based on combined-score clustering. Colour intensity reflects the frequency of the indicated event within each cohort. **(A)** Combined alteration score, defined as broad non-synonymous mutation frequency plus copy number amplification frequency. **(B)** Broad non-synonymous mutation frequency. **(C)** Copy number amplification frequency. **(D)** Expression over-frequency based on cohort-specific expression z-scores.

These patterns are illustrated in Figure 1, which summarizes the pan-cancer pharmacogenomic landscape across four complementary layers. The combined-score heatmap identified recurrent high-priority gene-cancer pairs, while the individual mutation, amplification, and overexpression panels showed that these signals were driven mainly by broad non-synonymous mutation and amplification rather than widespread transcriptional outliers alone. Consistent with the quantitative summaries, copy number deletion remained uncommon and is therefore shown separately in Supplementary Figure S1.

To formally assess whether alteration scores were more concentrated than expected under a simplified uniform-gene model, we performed 5,000 null simulations preserving the total number of broad non-synonymous mutation and copy number amplification events within each cancer type. The observed descriptive combined scores were more concentrated than expected under this null model. The observed global Gini index was 0.306 compared with a null mean of 0.248 (empirical p = 0.0002). The top 10 gene-cancer pairs accounted for 10.1% of the total combined score compared with a null mean of 7.8% (empirical p = 0.0002), while the top 30 accounted for 23.9% compared with a null mean of 20% (empirical p = 0.0002).

These findings support the interpretation that alteration scores were recurrent and non-uniform within the selected 40-gene pharmacogene panel under a simplified uniform-gene null model. However, this analysis does not demonstrate pharmacogene-specific enrichment relative to matched background genes and does not fully control for genomic location, aneuploidy, gene length, or tumor mutational burden. Detailed global and project-level null-model results are provided in Supplementary Tables S13, S14.

### Prioritization of recurrent pharmacogene alterations

**FIGURE 2:**
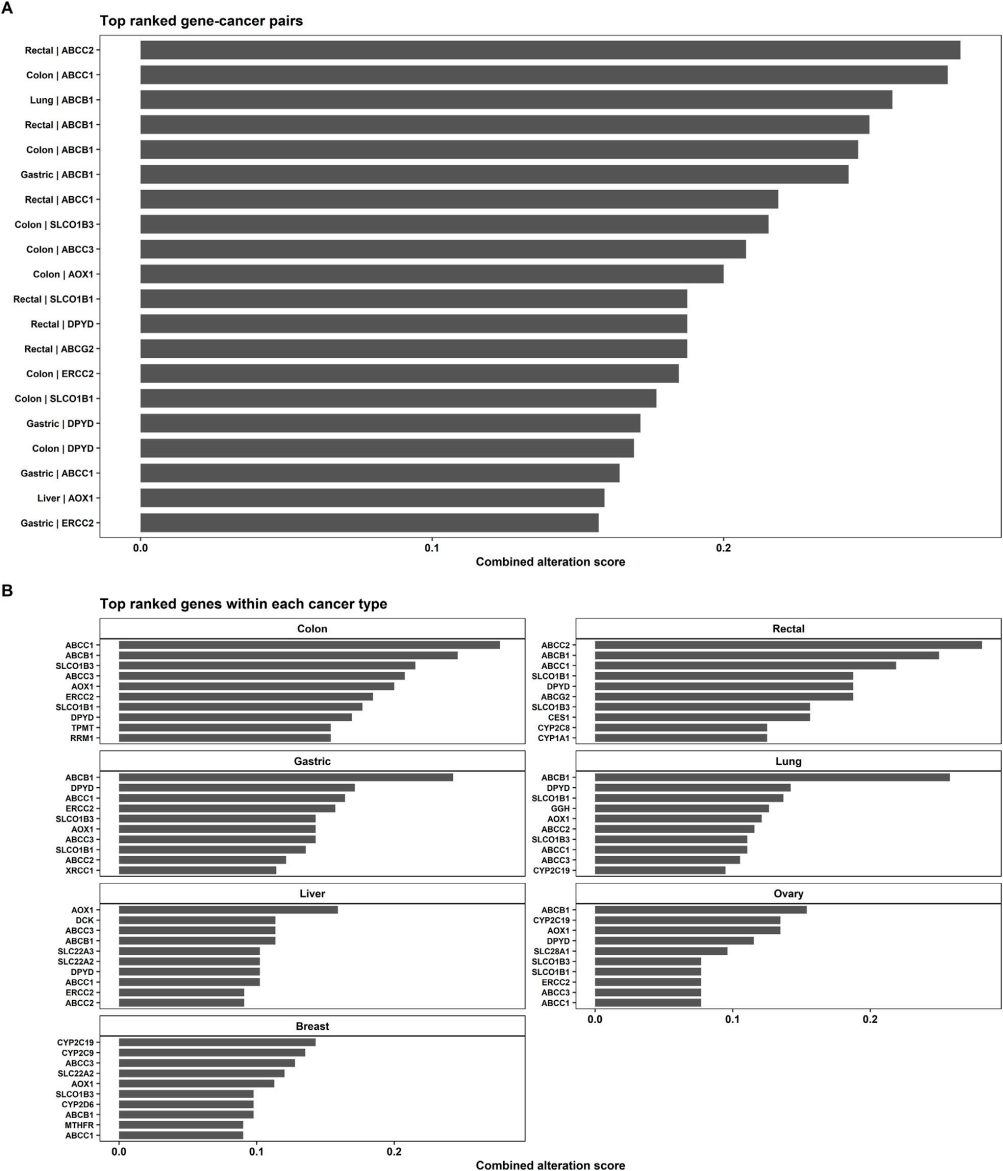
Prioritization of pharmacogenes by descriptive combined prioritization score. **(A)** Top gene-cancer pairs ranked by the descriptive combined score, defined as broad non-synonymous mutation frequency plus copy number amplification frequency. Each bar represents one pharmacogene within one cancer type. **(B)** Highest-ranking pharmacogenes within each cancer type using the same descriptive score.

To prioritize recurrent pharmacogene-cancer pairs, we calculated a descriptive combined prioritization score defined as broad non-synonymous mutation frequency plus copy number amplification frequency. This score was used for descriptive ranking only and was not interpreted as a functional or clinically actionable score. The ranking identified a limited subset of pharmacogenes that contributed disproportionately to the overall molecular alteration landscape (Figure 2; Table 3). The highest-ranking pair was *ABCC2* in TCGA-READ, with broad non-synonymous mutations observed in 8/32 patients (25%) and copy number amplification in 1/32 patients (3.1%), giving a combined prioritization score of 0.281. Because TCGA-READ was a relatively small cohort, this signal was interpreted cautiously. Other leading signals included *ABCC1* in TCGA-COAD, with broad non-synonymous mutations in 22/130 patients (16.9%) and amplification in 14/130 patients (10.8%; score 0.277), and *ABCB1* in TCGA-LUAD, with broad non-synonymous mutations in 36/190 patients (18.9%) and amplification in 13/190 patients (6.8%; score 0.258). *ABCB1* also appeared among the top-ranked genes in several cohorts, including LUAD, READ, COAD, and STAD, suggesting recurrent transporter-related alteration patterns across distinct cancer settings. Colon adenocarcinoma also showed several prominent high-ranking events, including *SLCO1B3*, *ABCC3*, *AOX1*, and *ERCC2*, consistent with the broader finding that alteration scores were concentrated in a subset of recurrent gene-cancer pairs rather than evenly distributed across the panel.

**TABLE 3:** Top pharmacogene-cancer pairs ranked by descriptive combined prioritization score.

Rank broad	Project	Gene	No. of patients	Broad non-synonymous n/N (%)	Broad non-synonymous 95% CI (%)	Truncating/ LoF-like n/N (%)	Truncating/ LoF-like 95% CI (%)	CNA amplification n/N (%)	CNA amplification 95% CI (%)	Broad combined score	Truncating combined score	Amp only score
1	TCGA-READ	ABCC2	32	8/32 (25.0)	13.3–42.1	2/32 (6.2)	1.7–20.1	1/32 (3.1)	0.6–15.7	0.281	0.094	0.031
2	TCGA-COAD	ABCC1	130	22/130 (16.9)	11.4–24.3	3/130 (2.3)	0.8–6.6	14/130 (10.8)	6.5–17.3	0.277	0.131	0.108
3	TCGA-LUAD	ABCB1	190	36/190 (18.9)	14.0–25.1	4/190 (2.1)	0.8–5.3	13/190 (6.8)	4.0–11.4	0.258	0.089	0.068
4	TCGA-READ	ABCB1	32	6/32 (18.8)	8.9–35.3	2/32 (6.2)	1.7–20.1	2/32 (6.2)	1.7–20.1	0.25	0.125	0.062
5	TCGA-COAD	ABCB1	130	23/130 (17.7)	12.1–25.2	4/130 (3.1)	1.2–7.6	9/130 (6.9)	3.7–12.6	0.246	0.1	0.069
6	TCGA-STAD	ABCB1	140	23/140 (16.4)	11.2–23.4	7/140 (5.0)	2.4–10.0	11/140 (7.9)	4.4–13.5	0.243	0.129	0.079
7	TCGA-READ	ABCC1	32	6/32 (18.8)	8.9–35.3	0/32 (0.0)	0.0–10.7	1/32 (3.1)	0.6–15.7	0.219	0.031	0.031
8	TCGA-COAD	SLCO1B3	130	16/130 (12.3)	7.7–19.1	6/130 (4.6)	2.1–9.7	12/130 (9.2)	5.4–15.4	0.215	0.138	0.092
9	TCGA-COAD	ABCC3	130	12/130 (9.2)	5.4–15.4	1/130 (0.8)	0.1–4.2	15/130 (11.5)	7.1–18.2	0.208	0.123	0.115
10	TCGA-COAD	AOX1	130	9/130 (6.9)	3.7–12.6	1/130 (0.8)	0.1–4.2	17/130 (13.1)	8.3–19.9	0.2	0.138	0.131
11	TCGA-READ	DPYD	32	6/32 (18.8)	8.9–35.3	1/32 (3.1)	0.6–15.7	0/32 (0.0)	0.0–10.7	0.188	0.031	0
12	TCGA-READ	ABCG2	32	4/32 (12.5)	5.0–28.1	1/32 (3.1)	0.6–15.7	2/32 (6.2)	1.7–20.1	0.188	0.094	0.062
13	TCGA-READ	SLCO1B1	32	4/32 (12.5)	5.0–28.1	1/32 (3.1)	0.6–15.7	2/32 (6.2)	1.7–20.1	0.188	0.094	0.062
14	TCGA-COAD	ERCC2	130	9/130 (6.9)	3.7–12.6	0/130 (0.0)	0.0–2.9	15/130 (11.5)	7.1–18.2	0.185	0.115	0.115
15	TCGA-COAD	SLCO1B1	130	11/130 (8.5)	4.8–14.5	4/130 (3.1)	1.2–7.6	12/130 (9.2)	5.4–15.4	0.177	0.123	0.092
16	TCGA-STAD	DPYD	140	21/140 (15.0)	10.0–21.8	10/140 (7.1)	3.9–12.6	3/140 (2.1)	0.7–6.1	0.171	0.093	0.021
17	TCGA-COAD	DPYD	130	18/130 (13.8)	8.9–20.8	3/130 (2.3)	0.8–6.6	4/130 (3.1)	1.2–7.6	0.169	0.054	0.031
18	TCGA-STAD	ABCC1	140	14/140 (10.0)	6.1–16.1	4/140 (2.9)	1.1–7.1	9/140 (6.4)	3.4–11.8	0.164	0.093	0.064
19	TCGA-LIHC	AOX1	88	8/88 (9.1)	4.7–16.9	1/88 (1.1)	0.2–6.2	6/88 (6.8)	3.2–14.1	0.159	0.08	0.068
20	TCGA-STAD	ERCC2	140	11/140 (7.9)	4.4–13.5	1/140 (0.7)	0.1–3.9	11/140 (7.9)	4.4–13.5	0.157	0.086	0.079
21	TCGA-READ	CES1	32	3/32 (9.4)	3.2–24.2	1/32 (3.1)	0.6–15.7	2/32 (6.2)	1.7–20.1	0.156	0.094	0.062
22	TCGA-READ	SLCO1B3	32	3/32 (9.4)	3.2–24.2	1/32 (3.1)	0.6–15.7	2/32 (6.2)	1.7–20.1	0.156	0.094	0.062
23	TCGA-OV	ABCB1	52	4/52 (7.7)	3.0–18.2	1/52 (1.9)	0.3–10.1	4/52 (7.7)	3.0–18.2	0.154	0.096	0.077
24	TCGA-COAD	TPMT	130	6/130 (4.6)	2.1–9.7	2/130 (1.5)	0.4–5.4	14/130 (10.8)	6.5–17.3	0.154	0.123	0.108
25	TCGA-COAD	RRM1	130	5/130 (3.8)	1.7–8.7	1/130 (0.8)	0.1–4.2	15/130 (11.5)	7.1–18.2	0.154	0.123	0.115
26	TCGA-COAD	XRCC1	130	5/130 (3.8)	1.7–8.7	0/130 (0.0)	0.0–2.9	15/130 (11.5)	7.1–18.2	0.154	0.115	0.115
27	TCGA-COAD	SLC29A1	130	4/130 (3.1)	1.2–7.6	1/130 (0.8)	0.1–4.2	16/130 (12.3)	7.7–19.1	0.154	0.131	0.123
28	TCGA-COAD	UGT1A1	130	4/130 (3.1)	1.2–7.6	0/130 (0.0)	0.0–2.9	16/130 (12.3)	7.7–19.1	0.154	0.123	0.123
29	TCGA-COAD	ERCC1	130	4/130 (3.1)	1.2–7.6	1/130 (0.8)	0.1–4.2	15/130 (11.5)	7.1–18.2	0.146	0.123	0.115
30	TCGA-STAD	AOX1	140	13/140 (9.3)	5.5–15.2	5/140 (3.6)	1.5–8.1	7/140 (5.0)	2.4–10.0	0.143	0.086	0.05

The descriptive combined prioritization score was defined as broad non-synonymous mutation frequency plus copy number amplification frequency. Broad non-synonymous mutations included missense, nonsense, splice-site, frameshift, translation-start-site, nonstop, and in-frame insertion/deletion variants. Truncating/LoF-like mutations included nonsense, frameshift, splice-site, and translation-start-site variants. CI, confidence interval; N, number of patients.

To assess the robustness of these rankings, we performed sensitivity analyses using stricter truncating/LoF-like mutation definitions and amplification-only rankings. The top 30 broad non-synonymous ranking showed 70% overlap with the truncating/LoF-like ranking. Small-cohort sensitivity analyses showed 73.3% overlap after excluding cohorts with fewer than 50 patients and 66.7% overlap after excluding cohorts with fewer than 100 patients. Amplification-only rankings were more stable, with 100% overlap after excluding cohorts with fewer than 50 patients and 83.3% overlap after excluding cohorts with fewer than 100 patients. These findings indicate that some READ-specific signals are unstable and should be considered exploratory, whereas several larger-cohort signals in TCGA-COAD, TCGA-LUAD, and TCGA-STAD remained among the top-ranked pharmacogene-cancer pairs. Full ranking-overlap and small-cohort sensitivity results are provided in Supplementary Tables S3–S5.

We further evaluated whether CNA-based prioritization was robust to alternative copy-number definitions by comparing the primary within-cohort z-score amplification calls with cBioPortal PanCancer Atlas GISTIC threshold copy-number calls. Across 280 gene-cancer pairs, mean amplification frequency was 4.7% using the z-score definition and 1% using strict GISTIC +2 amplification. Broad GISTIC gain/amplification calls were more frequent, averaging 19.7%, and therefore represented a less specific copy-number category. The top 30 z-score-based prioritization showed 63.3% overlap with the strict GISTIC +2 amplification ranking, indicating partial robustness to a more conservative CNA definition. Detailed GISTIC sensitivity results, including frequency comparisons, ranking overlap, and strict GISTIC +2 top-ranked pairs, are provided in Supplementary Tables S8–S10.

Score decomposition showed that the top 30 gene-cancer pairs captured both mutation-driven and CNA-driven signals, with a mean mutation contribution of 55.1% and a mean amplification contribution of 44.9%. Ranking overlap analyses further showed that the descriptive combined score overlapped by 63.3% with mutation-only rankings, 50% with CNA-only rankings, 73.3% with truncating/LoF-like plus CNA rankings, and 60% with cBioPortal GISTIC +2 amplification-based rankings. These results support the use of the score as a descriptive prioritization tool while reinforcing that it should not be interpreted as a functional, clinical, or treatment-response score. Score component decomposition and ranking overlap across score definitions are provided in Supplementary Tables S11, S12.

### Module-level pharmacogenomic heterogeneity

**FIGURE 3:**
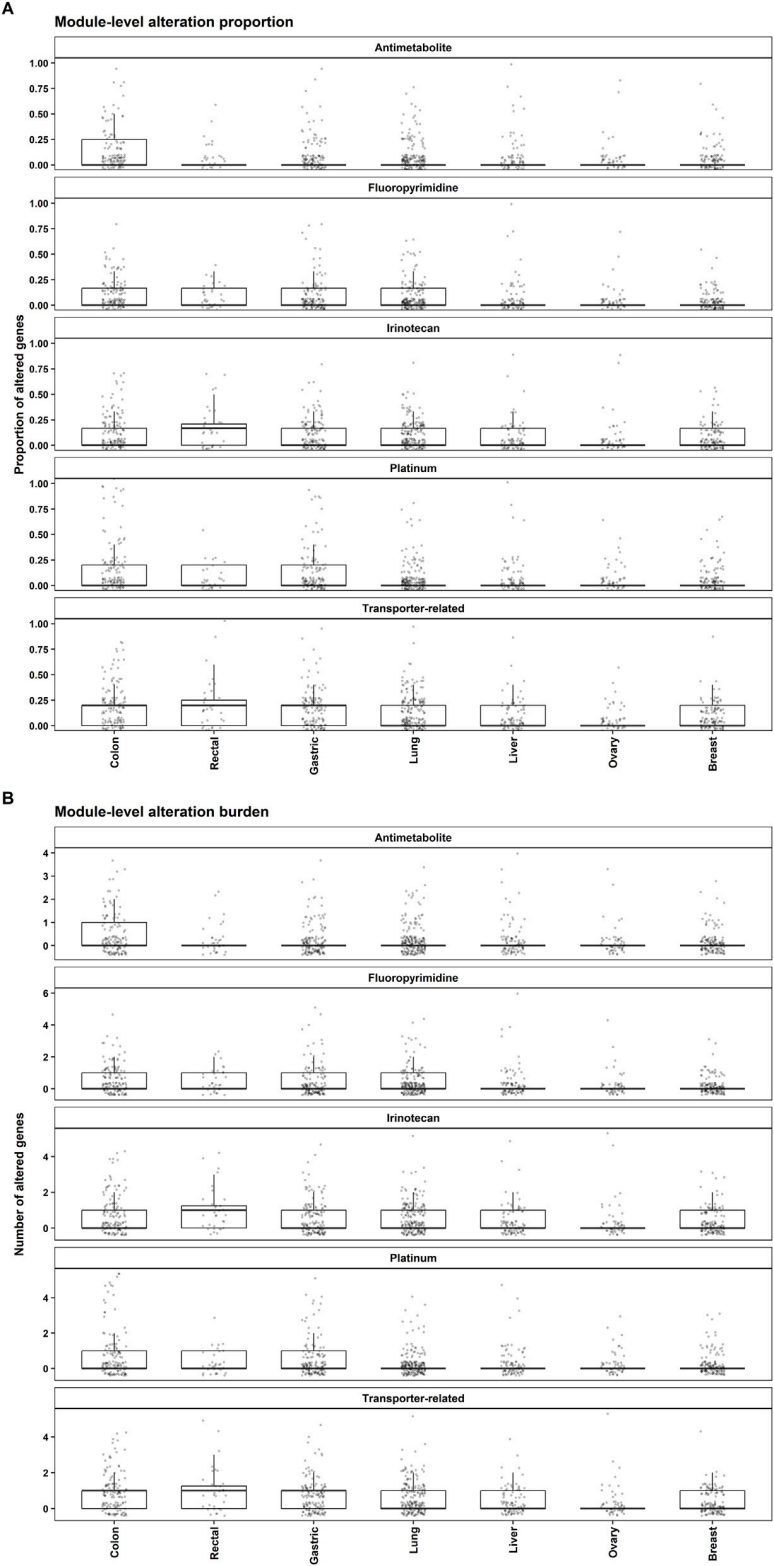
Module-level pharmacogenomic alteration patterns across cancer types. **(A)** Distribution of module alteration proportions across tumor samples, defined as the fraction of genes altered within each pharmacological module. **(B)** Distribution of module burdens across tumor samples, defined as the number of altered genes within each module. Each point represents an individual tumor sample, and boxplots summarize the within-cohort distribution. Modules include antimetabolite, fluoropyrimidine, irinotecan, platinum, and transporter-related pathways.

Although most individual pharmacogenes were altered at relatively modest frequencies, aggregation into functional pharmacological modules clarified broader pathway-level structure. At the sample level, most tumors showed low module alteration proportions and low module burdens, with medians of zero across modules, indicating that many tumors lacked integrated alterations within a given pathway. Nevertheless, a clear subset of tumors showed coordinated multi-gene disruption within specific modules, consistent with pathway-level pharmacogenomic heterogeneity rather than isolated single-gene events (Figure 3).

Across all samples, the transporter-related module showed the greatest overall disruption, with the highest mean alteration proportion and the highest average burden, followed by the irinotecan and platinum modules. The fluoropyrimidine and antimetabolite modules were altered less extensively overall, although they still showed broad inter-sample variation and occasional highly altered cases. These distributions indicate that the dominant pharmacogenomic signal in this dataset is centred on transport and drug-handling pathways, with additional contributions from DNA repair and antimetabolite-associated genes in more restricted subsets of tumors.

### Cohort-level module alteration patterns

**FIGURE 4:**
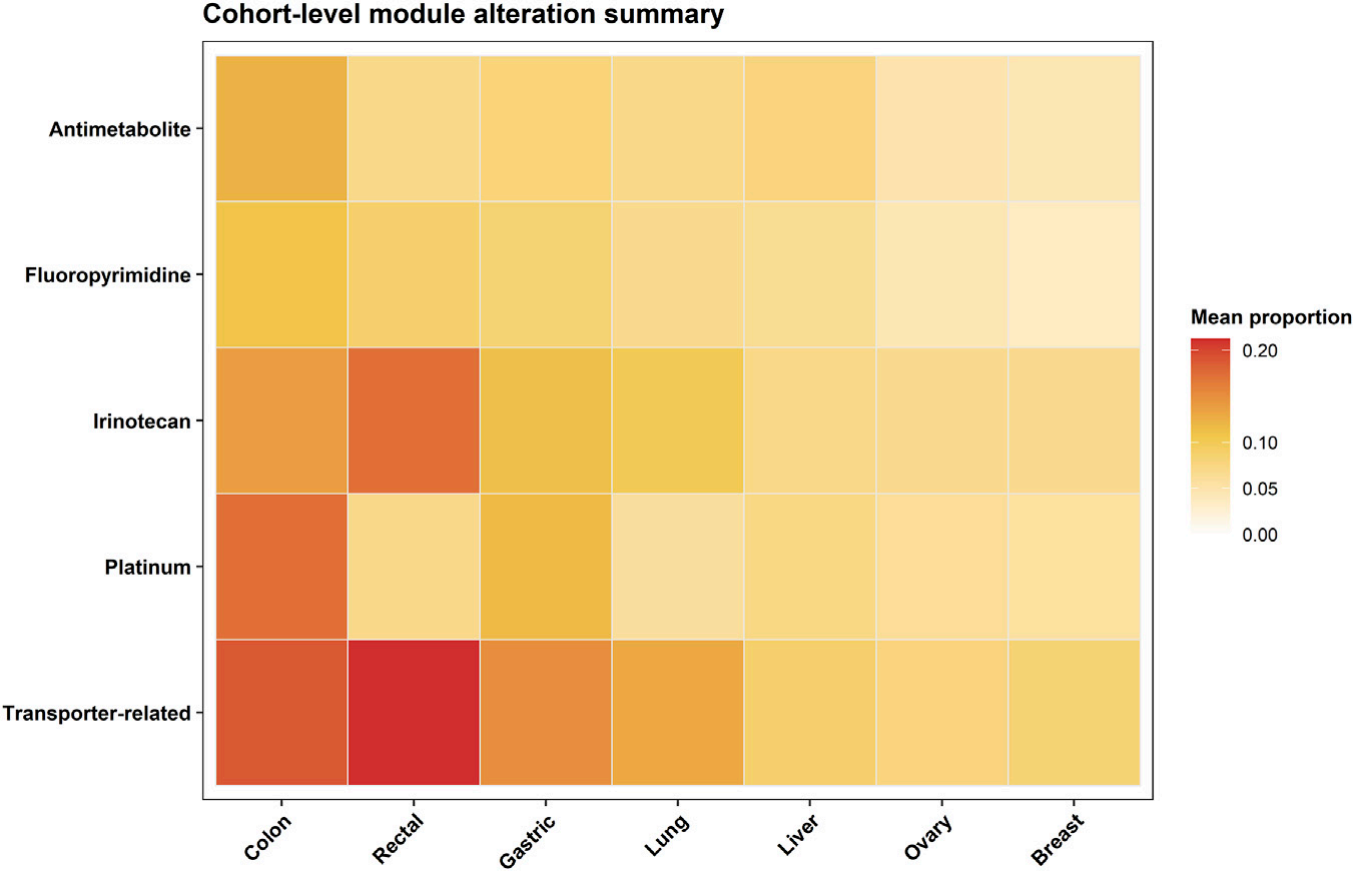
Cohort-level summary of module alteration patterns. Heatmap showing the mean proportion of altered genes within each pharmacological module across cancer types. Rows represent drug-related modules and columns represent tumor cohorts. Colour intensity reflects the average module-level alteration proportion within each cohort.

To compare pathway-level pharmacogenomic disruption across cancer types, mean module alteration proportions were summarized at the cohort level (Figure 4). This analysis again showed that the transporter-related module was the most consistently altered across cohorts, with particularly prominent values in TCGA-READ, TCGA-COAD, and TCGA-STAD. The irinotecan-associated module also showed comparatively high alteration levels across several cohorts, while the platinum module was especially prominent in TCGA-COAD and remained intermediate in STAD and LIHC.

By contrast, the fluoropyrimidine and antimetabolite modules showed lower average alteration proportions overall, although they still varied across tumor types and were not uniformly quiescent. These cohort-level patterns reinforce the gene-level findings and suggest that transporter-related pharmacogenomic variation represents the most consistently recurrent axis of disruption across the pan-cancer dataset.

### Systematic copy number-expression concordance across pharmacogenes

To evaluate whether recurrent genomic alterations were associated with transcriptional changes, we performed systematic CNA-expression concordance testing across all 280 pharmacogene-cancer pairs. All tested pairs had complete copy number and expression data. Spearman correlation analysis identified 64 gene-cancer pairs with significant positive CNA-expression associations after FDR correction, and linear models identified 68 significant associations. Strict amplification was associated with higher expression than neutral copy number state in 21 gene-cancer pairs after FDR correction, whereas no strict deletion-lower-expression association survived correction. The full systematic CNA-expression association results are provided in Supplementary Table S6 and summarized visually in Supplementary Figure S2.

**FIGURE 5:**
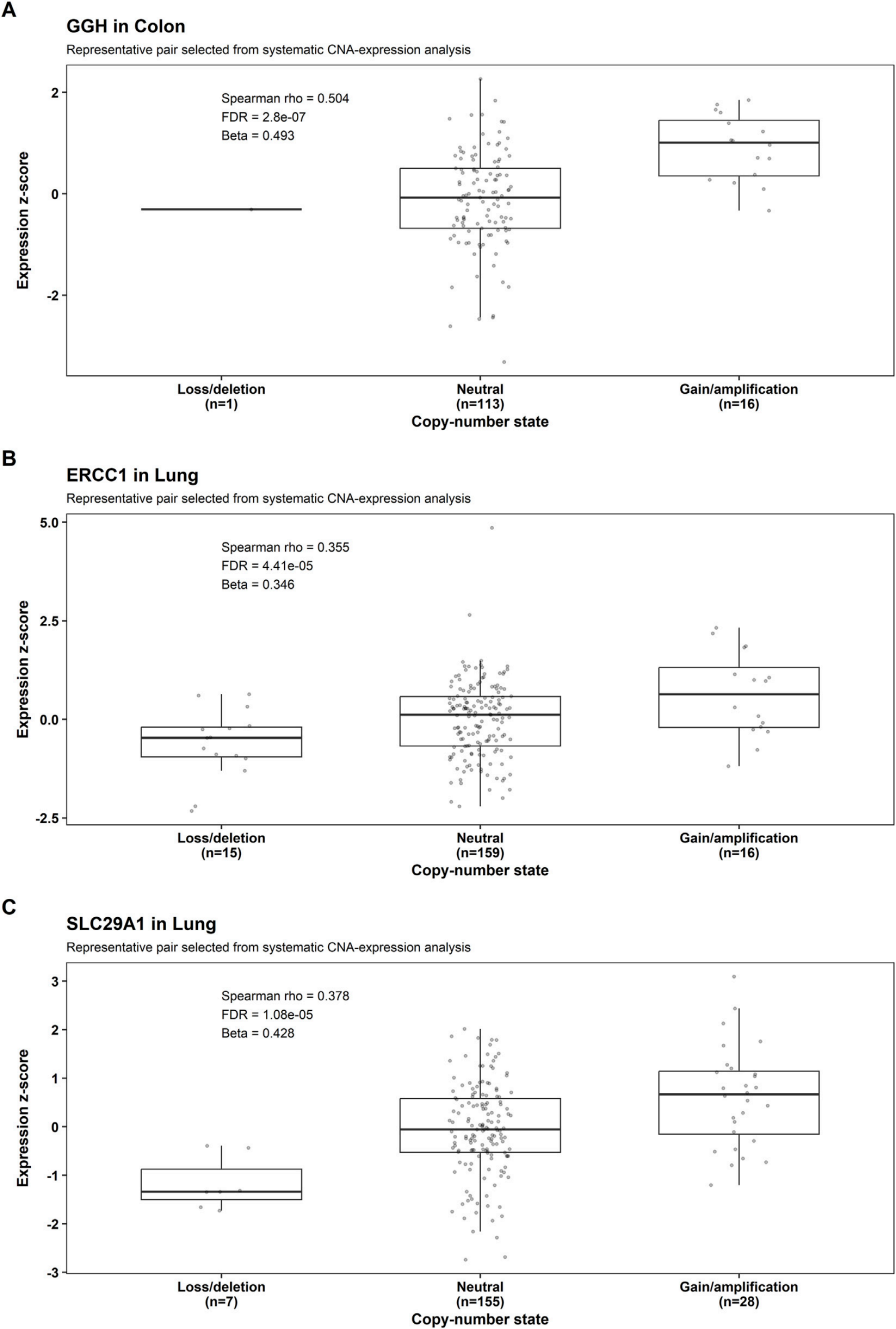
Representative copy number-associated expression patterns selected from systematic CNA-expression analysis. Expression z-scores are shown across copy number states for selected pharmacogene-cancer pairs identified from systematic testing across 280 gene-cancer pairs. **(A)** GGH in TCGA-COAD. **(B)** ERCC1 in TCGA-LUAD. **(C)** SLC29A1 in TCGA-LUAD. Group sample sizes are shown on the x-axis. Spearman correlation coefficients, FDR-adjusted p-values, and linear-model beta coefficients are shown within each panel. GGH in TCGA-COAD and SLC29A1 in TCGA-LUAD showed significant positive continuous CNA-expression associations and significant strict amplification-versus-neutral expression differences. ERCC1 in TCGA-LUAD showed a significant continuous CNA-expression association, but the strict amplification-versus-neutral comparison was not statistically significant after correction. These examples represent selected significant CNA-expression associations and should not be interpreted as direct functional validation.

Representative examples selected from this systematic analysis are shown in Figure 5. *GGH* in TCGA-COAD and *SLC29A1* in TCGA-LUAD showed significant positive continuous CNA-expression associations and higher expression in the strict amplification group compared with the neutral copy number group. *ERCC1* in TCGA-LUAD showed a significant positive continuous CNA-expression association, but the strict amplification-versus-neutral comparison was not statistically significant after correction. Therefore, the *ERCC1*-LUAD result should be interpreted as evidence of a continuous copy number-expression association rather than as a confirmed strict amplification-associated expression difference. These examples support the presence of dosage-associated expression patterns for selected pharmacogene-cancer pairs, although they do not establish direct functional or treatment-response effects.

### Association of pharmacogenomic module alteration with overall survival

We next performed exploratory treatment-agnostic survival analyses to assess whether module-level pharmacogene alteration proportions were associated with overall survival. These analyses were restricted to 733 patients with complete survival and clinical covariate data. No statistically significant associations were observed for any module (Table 4). Hazard ratios were close to unity across all modules, and all confidence intervals included 1.0. Because treatment exposure and drug-specific response data were unavailable, these analyses should not be interpreted as tests of pharmacogenomic predictiveness.

**TABLE 4:** Exploratory association between pharmacogenomic module proportion and overall survival. Cox proportional hazards models used module proportion as a continuous predictor and were adjusted for age and sex, with cancer type included as a stratification factor.

Module	Hazard ratio	95% CI	p-value
Fluoropyrimidine	1.05	0.93–1.19	0.454
Irinotecan	0.97	0.85–1.11	0.635
Platinum	1.07	0.94–1.22	0.282
Antimetabolite	1.00	0.87–1.14	0.970
Transporter-related module	0.95	0.82–1.09	0.430

N, number of cases; CI, confidence interval.

## Discussion

This study provides a TCGA pan-cancer view of somatic alterations in pharmacogenes using an integrated multi-omics framework. The analysis showed that pharmacogene alterations were generally infrequent at the individual gene level but displayed recurrent, non-uniform patterns across cancer types and pharmacological modules. A uniform-gene null model supported this interpretation by showing that descriptive combined scores were more concentrated than expected when mutation and amplification events were randomly redistributed across the 40-gene panel within each cancer type. These findings should be interpreted as an exploratory molecular map rather than evidence of direct pharmacogenomic actionability, because harmonized treatment exposure, response, and toxicity data were not available. These findings remain relevant because pharmacogenes are often not classic oncogenic drivers, yet they may influence tumor-associated drug handling through effects on transport, metabolism, and DNA damage response (Tremmel et al., 2025).

A central observation of this study is that pharmacogenomic alterations were individually infrequent whereas aggregation into pathway-level modules revealed clearer module-level patterns. This supports the idea that pharmacogenomic heterogeneity in tumors may be better captured at the level of coordinated pharmacological processes rather than isolated genes, which is biologically plausible because uptake, metabolism, efflux, and DNA repair together shape intracellular drug exposure and treatment sensitivity (Hyman et al., 2017; Letai, 2017). A pathway-oriented view is also more compatible with the way drugs act *in vivo*, where uptake, activation, inactivation, repair, and efflux collectively shape response (Tang et al., 2024). Therefore, the present analysis extends prior precision-oncology concepts by focusing not on canonical driver targets, but on a pharmacogene-centred alteration map that may inform future treatment-specific studies.

Copy number amplification emerged as a prominent alteration type within the pharmacogene panel across the analysed cancer types. This finding is consistent with established models of cancer drug resistance, particularly for transporter-associated genes, where increased gene dosage may contribute to higher transcript abundance and altered intracellular drug disposition. The prominence of transporter-related alterations in the present study is consistent with the long-recognized role of ABC transporters in multidrug resistance and with broader evidence that these proteins may also contribute to tumor aggressiveness beyond classical efflux-based resistance alone (Robey et al., 2018; Orlando and Liao, 2020).

The systematic CNA-expression analysis strengthened the interpretation of selected copy number events by showing that 64 of 280 pharmacogene-cancer pairs had significant positive copy number-expression associations after FDR correction. These findings support dosage-associated transcriptional effects for a subset of pharmacogenes, but they should not be interpreted as direct functional validation or evidence of treatment response. Importantly, the aggregation of gene-level alterations into functional modules revealed pharmacologically interpretable module-level patterns. Although these examples do not constitute direct functional validation, the observed direction of effect is consistent with dosage-associated transcriptional regulation. This is particularly relevant for *ERCC1*, given the established relationship between DNA repair capacity and platinum sensitivity, including evidence that *ERCC1* status can influence benefit from cisplatin-based therapy (Olaussen et al., 2006; Lord and Ashworth, 2012; Huang and Zhou, 2021). Similarly, the relevance of *SLC29A1* is biologically plausible because nucleoside transporter activity is central to gemcitabine influx, and human equilibrative nucleoside transporter 1 (hENT1)-related expression patterns have been linked to outcomes in gemcitabine-treated cancers (Mackey et al., 1998; Giovannetti et al., 2006; Xi et al., 2022).

The absence of significant associations between pharmacogene module proportion and overall survival should be interpreted cautiously. Overall survival is a treatment-agnostic endpoint and is not ideal for evaluating pharmacogenomic effects in the absence of harmonized treatment exposure. Pharmacogenomic features may be more relevant as predictors of drug response, resistance, or toxicity than as treatment-independent prognostic markers (Roden et al., 2019; Normanno et al., 2022). Therefore, the negative survival findings should be viewed as exploratory and do not exclude potential treatment-specific relevance. In heterogeneous retrospective cohorts without harmonized treatment exposure, their impact on survival can therefore be diluted. From this perspective, the negative survival result in the present study is compatible with the broader distinction between prognostic and predictive biomarkers emphasized in recent biomarker and precision-oncology literature (Lawler et al., 2024; Marletta et al., 2024).

The findings also have implications for biomarker strategy. From a translational standpoint, these findings support the idea that tumor pharmacogenomics may complement, rather than replace, canonical driver-focused oncology. Current precision-oncology frameworks increasingly emphasize broader molecular profiling and biomarker-guided patient selection, especially in settings where classic targetable alterations are absent or insufficient to explain therapeutic variability (Hyman et al., 2017; Letai, 2017). A pharmacogene-centered multi-omics framework fits well within this direction because it captures tumor-associated factors that may influence the effectiveness of standard cytotoxic and targeted agents, even when those genes are not directly actionable in the classic sense (Iorio et al., 2016).

This study has several limitations. First, the analysis was restricted to a curated 40-gene pharmacogene panel and therefore does not capture the full breadth of tumor-associated determinants of drug response. Second, harmonized treatment exposure, response, toxicity, dose modification, and progression-free survival data were not available, limiting direct assessment of pharmacogenomic predictiveness. Third, some cancer-specific cohorts, particularly TCGA-READ, were relatively small, making some high-percentage findings unstable. In addition, the null-model analysis tested score concentration against a simplified uniform-gene background and therefore supports non-uniformity within the selected 40-gene panel, but it does not demonstrate pharmacogene-specific enrichment relative to matched background genes. The analysis also does not fully control for genomic location, aneuploidy, gene length, tumor mutational burden, tumor purity, ploidy, batch effects, or other cancer-type-specific technical factors. Tumor purity, ploidy, batch effects, and cancer-type-specific technical factors were not directly modelled, and these factors may influence some copy number and expression-based estimates. Fifth, the study was based on TCGA data only, and independent validation in treatment-annotated or pharmacogenomic drug-response datasets will be required. Finally, although systematic CNA-expression testing was performed, expression concordance does not establish direct functional or therapeutic consequences. These limitations mean that the present findings should be interpreted as an exploratory descriptive map of tumor pharmacogene variation rather than as a direct clinical-response study. At the same time, the use of integrated mutation, copy-number, and transcriptomic data remains a strength, because multi-omics approaches are increasingly viewed as important for capturing clinically relevant heterogeneity that may be missed by single-layer analyses.

Future work should extend this approach in three directions. The first is clinical: linking pharmacogene modules to treatment-specific outcomes, toxicity, and resistance endpoints. The second is analytical: performing broader cross-layer concordance analyses across all genes rather than representative examples only. The third is translational: integrating tumor pharmacogenomics with germline pharmacogenomic information, which is increasingly recognized as an important step toward fuller cancer pharmacotherapy personalisation. Multi-omics and machine learning approaches may be useful here, but their value will depend on careful clinical direction paired with robust treatment annotation, transparent modelling strategies, and external validation.

## Conclusion

This study provides an exploratory TCGA pan-cancer map of somatic pharmacogene alterations across seven cancer types. Individual pharmacogene alterations were generally infrequent, but recurrent and non-uniform gene- and module-level patterns were observed across therapeutic pathways. Null-model analysis showed that descriptive alteration scores were more concentrated than expected under a simplified uniform-gene background. Copy number amplification represented a prominent alteration type, and systematic CNA-expression testing identified multiple gene-cancer pairs with dosage-associated expression patterns. Module-level pharmacogene alteration was not associated with overall survival in treatment-agnostic exploratory analyses. These findings generate hypotheses for future studies linking tumor pharmacogene alterations to treatment-specific response, toxicity, and external pharmacogenomic validation datasets.

## Data Availability

The original contributions presented in the study are included in the article/Supplementary Material, further inquiries can be directed to the corresponding author.

## Supplementary material

SUPPLEMENTARY FIGURE S1 Pan-cancer heatmap of copy number deletion frequency across pharmacogenes. Rows represent pharmacogenes and columns represent cancer types. Colour intensity reflects the frequency of strict copy number deletion events within each cohort. Deletion frequencies were generally low across the panel, supporting the main-text focus on amplification rather than deletion.

SUPPLEMENTARY FIGURE S2 Systematic CNA-expression concordance across pharmacogene-cancer pairs. Heatmap showing Spearman correlation coefficients between copy number z-score and expression z-score across 280 pharmacogene-cancer pairs. Positive values indicate higher expression with increasing copy number. Significant associations after FDR correction are reported in Supplementary Table S6.

SUPPLEMENTARY FIGURE S3 Global score concentration under a uniform-gene null model. Distribution of Gini index values from 5,000 null simulations in which broad non-synonymous mutation and copy number amplification events were randomly redistributed across the 40-gene pharmacogene panel within each cancer type while preserving the total number of events per cancer type. The vertical line indicates the observed Gini index.

SUPPLEMENTARY FIGURE S4 Top-10 score share under a uniform-gene null model. Distribution of the fraction of total descriptive combined score contributed by the top 10 gene-cancer pairs across 5,000 null simulations. The vertical line indicates the observed top-10 score share.
