# Supplementary material for: Pan-cancer multi-omics analysis of pharmacogenomic alterations
Source: Front Bioinform. 2026 Jul 22;6:1858518. doi: 10.3389/fbinf.2026.1858518 (PMC13437544; doi:10.3389/fbinf.2026.1858518)
Supplement: Supplementary file 1 [file Supplementaryfile1.docx]

**Supplementary Figure S1**

**Supplementary Figure S2**

**Supplementary Figure S3**

**Supplementary Figure S4**
